# Treatment Length and External Iliac Artery Extension Are Associated with Increased Aortic Stiffness After Endovascular Aortic Repair: A Prospective, Monocentric, Single-Arm Study

**DOI:** 10.3390/biomedicines13061279

**Published:** 2025-05-23

**Authors:** Manolis Abatzis-Papadopoulos, Konstantinos Tigkiropoulos, Spyridon Nikas, Christina Antza, Christina Alexou, Anthi-Maria Lazaridi, Kyriakos Stavridis, Vasilios Kotsis, Ioannis Lazaridis, Nikolaos Saratzis

**Affiliations:** 1 Surgical Department, General Hospital of Katerini, 60100 Katerini, Greece; 2 Vascular Unit, 1st University Surgical Department, Papageorgiou General Hospital, School of Medicine, Aristotle University of Thessaloniki, 56403 Thessaloniki, Greece; kostastig@yahoo.com (K.T.); kstavridis17@yahoo.gr (K.S.); drlazaridis@yahoo.gr (I.L.); nicos_saratzis@yahoo.com (N.S.); 3 Radiology Department, Papageorgiou General Hospital, 56403 Thessaloniki, Greece; sncorfu@hotmail.com; 4 3rd University Department of Internal Medicine, Papageorgiou General Hospital, School of Medicine, Aristotle University of Thessaloniki, 56403 Thessaloniki, Greece; kris-antza@hotmail.com (C.A.); vkotsis@auth.gr (V.K.); 5 Cardiothoracic Surgery Department, Papanikolaou General Hospital, 57010 Thessaloniki, Greece; christinaalexou@gmail.com; 6 Primary Care Unit of Servion, General Hospitalof Kozani, 50100 Kozani, Greece; anthilazaridi@gmail.com

**Keywords:** abdominal aortic aneurysm, pulse wave velocity, aortic compliance, aortic stiffness, endovascular aortic repair, aortic endograft

## Abstract

**Background/Objectives**: Aortic stiffness is a strong independent factor in cardiovascular outcomes. The method of choice for evaluating aortic stiffness is the measurement of aortic pulse wave velocity (PWV). Endovascular aortic repair (EVAR) increases aortic rigidity and thus aortic stiffness. The aim of this study is to investigate the correlation between endograft length and post-operative increases in PWV in patients with abdominal aortic aneurysms (AAAs) subjected to EVAR. **Methods**: A prospective observational study enrolling 107 patients from February to December 2025 was conducted. Patient demographics and comorbidities were recorded. The length of the endografts was calculated by studying computed tomography angiograms (CTAs) and digital subtraction angiographies (DSAs) of the patients. PWV was measured pre-operatively and post-operatively during the first 24 h after EVAR, and the difference in PWV (dPWV) was calculated. **Results**: The mean age of the patients was 72 ± 7.5 years, and 93.5% of them were males. The mean transverse AAA diameter was 5.7 ± 1.1 mm, and the mean endograft length was 169.7 ± 26.9 mm. An extension to the external iliac artery was deployed in 10 patients (9.3%). A strong positive correlation was observed between dPWV and endograft length, indicating that each additional 1 mm in graft length corresponded to a 0.541% increase in dPWV. Patients with an extension to external iliac arteries exhibited a significantly higher mean dPWV (9.95 ± 2.08% vs. 27.12% ± 12.15%, t = −4.463, *p* = 0.002). No statistically significant differences in dPWV between the different endograft types were found (*p* = 0.74). **Conclusions**: Endograft length is strongly related to PWV elevation during the immediate post-operative time after EVAR, especially when the endograft is extended to the external iliac arteries.

## 1. Introduction

Abdominal aortic aneurysms (AAAs) affect 2–8% of males over the age of 65 [1]. The basic pathophysiologic mechanism of AAA development is the degradation of the tunica media by a proteolytic process, which leads to the elimination of elastin from the media [2]. The vast majority of AAAs are asymptomatic and diagnosed incidentally by various imaging techniques. As AAAs expand, they cause symptoms like pain due to compression of nearby organs, lower extremity ischemia due to peripheral embolization or thrombosis, or even rupture, causing hemodynamic instability. Computed tomography (CT) presents high sensitivity in diagnosing AAAs, although ultrasound is used for the screening of patients. According to the European Society of Vascular Surgery (ESVS) guidelines, patients should be managed surgically when their AAAs surpass the threshold of 5.5 cm and 5 cm in diameter in men and women, respectively. Open surgical repair (OSR) is considered the method of choice for elective management of AAAs in young, fit patients [3]. However, endovascular aortic repairs (EVARs) have now exceeded and outnumbered open surgical repairs (OSRs) of abdominal aortic aneurysms (AAAs) globally [4]. This trend is supported by the fact that patients subjected to EVAR, especially elderly patients and ruptured AAA patients, present lower morbidity and mortality rates compared with patients subjected to OSR post-operatively according to the European Society of Vascular Surgery (ESVS) guidelines [3].

Available commercial endografts consist of a metallic skeleton made of nitinol or other metal alloy stents, and their covered parts are composed of fabrics made of either Dacron polyester or expanded polytetrafluoroethylene (ePTFE). Consequently, the implantation of endografts during EVAR causes an increase in aortic rigidity and, hence, aortic stiffness, which results in decreasing abdominal aorta compliance [5].

The method of choice (‘gold-standard’) for assessing aortic stiffness and compliance is the measurement of the aortic pulse wave velocity (PWV) [6]. Aortic stiffness has been established as an independent predictor of cardiovascular mortality in hypertensive, diabetic, and end-stage renal disease patients [7,8,9]. Furthermore, several studies demonstrate the association of increased PWV with cardiovascular mortality in the general population as well [10]. As a result, aortic stiffness is strongly and positively associated with adverse cardiovascular outcomes and is proven to be an independent predictor of cardiovascular mortality [11].

Several studies have demonstrated the probable deleterious effect of EVAR on various organs, including the heart and the kidneys [12,13,14]. In the literature, various studies have been published investigating the properties of endografts, which are correlated with the increase in aortic stiffness and PWV. Although the association between endograft length and PWV increase has been studied in patients with thoracic aortic aneurysms subjected to thoracic endovascular aortic repair (TEVAR) by Hori et al. [15], studies investigating this association in patients with AAAs have not been published yet to the best of our knowledge. The aim of this study is to investigate the association between endograft length and the post-operative increase in PWV in patients with AAAs subjected to EVAR. Moreover, this study intended to investigate other endograft properties associated with PWV and aortic stiffness increases. Investigating various properties of the endografts associated with the increases in PWV and aortic stiffness and anticipating cardiovascular events induced by increased aortic stiffness related to endograft implantation could provide the enhancement of these properties, meaning that endografts’ impact on the native aortic wall can be ameliorated.

## 2. Materials and Methods

### 2.1. Study Design

This was a prospective, observational, monocentric, and single-arm study. Patients meeting the inclusion criteria were recruited in our study in a consecutive way from February 2025 to December 2025. An informed written consent was signed by all patients participating in our study. A detailed medical history of each patient was recorded. Pre-operatively, a computed tomography angiogram (CTA) was conducted for every patient, so that the anatomical characteristics of their AAA could be recorded. Digital subtraction angiography (DSA) images were collected during the EVAR procedure. Further details regarding the operation (operation time, radiation time, radiation dose, and contrast quantity) and any complications during the operation and the immediate 48 h post-operative period were recorded. PWV was measured pre-operatively and post-operatively during the first 24 h after EVAR, and the change in PWV (dPWV) was calculated and expressed as an absolute number and as a percentage.

### 2.2. Sample Size

We conducted a power analysis regarding the primary outcome of the effect of endograft length on the PWV. Hori et al.’s study was used for the expected effect, as graft length is an independent prognostic factor for PWV increase. We used a fixed model single regression coefficient for our power analysis to incorporate the findings related to effect size suggested in the study by Hori et al. [15]. A *p*-value of less than 0.05 is considered to be statistically significant, while the power level required is at least 80%, using the relevant literature. By incorporating a maximum of 10 confounders in the model, we found that a sample size of 107 patients is required. No withdrawals of patients were considered in this estimation, while in the case that any patient was excluded according to the inclusion/exclusion criteria, an additional patient was to be prospectively enrolled.

### 2.3. Inclusion/Exclusion Criteria

Patients were eligible to be recruited in our study when they met the following criteria: adult male or female patients with infrarenal AAAs exclusively who were subjected to elective EVAR with aortobiiliac endografts. If required, the endograft was extended to the external iliac arteries during EVAR. The threshold of AAA diameter for EVAR was a diameter of ≥5.5 cm in men and ≥5 cm in women according to the ESVS guidelines [2] (Table 1).

The exclusion criteria were the following: patients suffering from ruptured, inflammatory, or mycotic AAAs, patients with complex AAAs, such as juxtarenal or thoracoabdominal aortic aneurysms, patients managed with chimney EVAR (ChEVAR), fenestrated EVAR (FEVAR), or branched EVAR (BEVAR) procedures, patients managed with EVAR using aortouniiliac devices or straight tube endografts, patients with connective tissue disorders, patients whose PWV measurement could be biased like in end-stage renal disease patients, patients suffering from severe atherosclerotic disease of the brachiocephalic and/or right common carotid and/or internal carotid and/or right common iliac and/or external iliac and/or right common femoral arteries with severe hemodynamic changes, patients having already been managed for their atherosclerotic disease with operations (open and/or endovascular) on these arteries, and patients with previous repairs of the aorta subjected to open and/or endovascular operations on the ascending aorta and/or the aortic arch and/or the descending thoracic and/or the abdominal aorta (Table 1).

### 2.4. Definition of Variables

**Figure 1:**
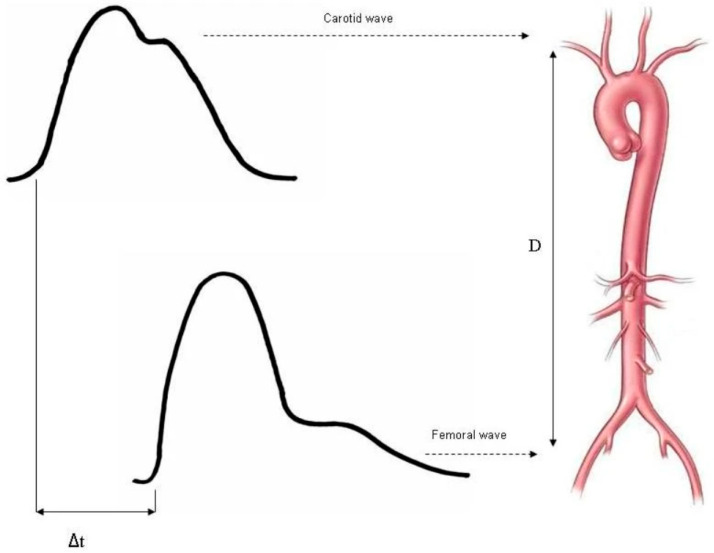
PWV measurement using the distance (D) and the transit time (Δt) between the feet of the waveforms of the right common carotid and femoral arteries [18].

The primary endpoint of this study was the correlation between the lengths of various endografts implanted during EVAR and the increase in aortic stiffness measured by post-operative PWV compared to the pre-operative PWV. Pre-operative CTAs of patients were studied with the 3mensio vascular workstation 10.4 (Pie Medical Imaging BV, Philipsweg 1, 6227 AJ Maastricht, The Netherlands) to define the centerline of the aortic lumen [16]. This allowed for the calculation of the distance between the lowest renal artery and the right common iliac bifurcation, as the covered part of the endografts was deployed just below the lowest renal artery, and the iliac legs were landed on the common iliac arteries or on the external iliac arteries if needed. The length of the endograft was calculated by studying the pre-operative CTA analysis with 3mensio software, the digital subtraction angiographies (DSAs) performed during the EVAR procedure, and the radiopaque markers of the endografts. PWV measurement was conducted using the Complior system (Alam Medical, 69 B rue de Mallacombe 38070 Saint Quentin Fallavier France) and by following the recommendations of the Artery Society, the European Society of Hypertension Working Group on Vascular Structure and Function, and the European Network for Noninvasive Investigation of Large Arteries [17]. The method usually applied for calculating PWV uses the foot-to-foot velocities from various waveforms [6]. The waveforms of the right common carotid and the right femoral artery are usually transcutaneously acquired by placing transcutaneous transducers above the palpable pulses of the right common carotid and right common femoral arteries. The device detects the transit time (defined as Δt) between the waveforms of these two arteries, while the researchers measure the distance between the right common carotid and the right common femoral arteries (defined as D) of the patients using a centimeter tape. PWV was calculated automatically by the device and was the result of the division of the distance measured by the researchers by the transit time provided by the device (PWV = D/Δt) [17]. The averages of two pre- and two post-operative PWV measurements were used to calculate pre- and post-operative PWV, respectively. Figure 1 presents the method of PWV measurement using the foot-to-foot method.

The secondary endpoints of this study included the correlation of endograft material related to aortic stiffness and the PWV increase by separating the EVAR patients into three groups: (i) patients having received a Dacron polyester (D) endograft, (ii) patients having received an ePTFE endograft with suprarenal fixation (Ps), and (iii) patients having received an ePTFE endograft with infrarenal fixation (Pi). Further secondary endpoints of our study included the intraoperative and immediate post-operative complications within 48 h after EVAR.

All EVAR procedures conducted on patients included in this study were elective and were performed in a fully equipped operating suite with a radiolucent table under fluoroscopic guidance of a portable C-arm device. In order to decrease the risk of bleeding during EVAR, patients receiving acenocoumarol were advised to discontinue the medicine five days before EVAR, and bridging therapy with low-molecular-weight heparin (LMWH) was prescribed if needed according to the pre-operative recording of anticoagulation. Similarly, patients receiving new oral anticoagulants were advised to discontinue the medication for at least two days prior to EVAR according to the type of anticoagulant they were receiving, their comorbidities, and their hemorrhagic risk. Patients were administered either general or regional anesthesia during EVAR. The type of anesthesia and any relevant complications were recorded. EVAR procedures were conducted through the common femoral arteries, and surgical access through them was gained after standard surgical exposure with surgical cut-down and arteriotomy. All patients were administered antibiotic prophylaxis prior to EVAR. During the procedure, anticoagulation was achieved by means of intravenous unfractioned heparin administration at a dosage of 100 IU/kg body weight. Acute clotting time was recorded during EVAR, and an additional heparin dose was administered when required. All medications administered to the patients during the perioperative period were recorded. Routinely and under fluoroscopic guidance, a stiff 0.035 wire was advanced up to the aortic arch through the ipsilateral vascular access. A pigtail angiographic catheter was advanced retrogradely from the contralateral vascular access through the respective common femoral artery to a level just proximal to the renal arteries. A DSA was conducted using this pigtail catheter. Iobitridol with a concentration of 350 mg/L was used as a contrast medium, apart from the patients presenting an absolute contraindication. The main body of the endograft was deployed with its covered part left just below the lowest renal artery. Bifurcations of the aorta and common iliac arteries, including hypogastric arteries, were marked through a DSA. The contralateral gate of the main body of the endograft was cannulated using a catheter advanced through the contralateral vascular access retrogradely in order to secure contralateral wire access. A second stiff 0.035 wire was then advanced up to the aortic arch from the contralateral vascular access via the respective common femoral artery. The extension limbs were then deployed and landed on the common iliac arteries by taking care to avoid coverage of the hypogastric artery origins. Balloon angioplasty was performed at the docking zone of the main body and along the iliac limbs. If there was an intention to perform any additional procedures, such as embolization of the hypogastric vessels, these were usually performed prior to EVAR. These procedures, along with any extension of the endografts to the external iliac arteries, were recorded. After the completion of the endovascular procedure, a final DSA was conducted in order to check technical success by verifying exclusion of the aneurysm, patency of the renal arteries, and iliac limbs. Subcutaneous tissues and skin were closed in the standard way. Necessary DSA images were gathered to measure the length of the endograft deployed. Patients were administered lifelong single antiplatelet therapy, except for patients receiving oral anticoagulants prior to EVAR, who were discharged on LMWH for a short period, followed by bridging therapy with their pre-operative oral anticoagulants.

Primary technical success was defined as successful access to the arterial system, successful deployment of the endograft with secure proximal and distal sealing, fixation of the attachment devices, demonstrating safe and effective exclusion of the AAA without type I or III endoleaks, and a patent endograft without significant twists, kinks, or obstruction and without the need for an additional secondary surgical procedure. Secondary technical success was defined as the successful completion of EVAR with an additional secondary surgical procedure during the initial operation after initial deployment of the endograft without the need for open conversion.

### 2.5. Data Collection

All relevant data were recorded in spreadsheets regarding demographics of the patients, their medical and surgical history, family medical history, comorbidities, medications, smoking habits, alcohol consumption, anatomical characteristics of AAA, the length and other properties of the endografts, pre- and post-operative PWV measurements, operative details, technical success, and any adverse events/complications.

### 2.6. Statistical Synthesis and Analysis

Categorical variables were described using counts and percentages, while continuous variables were summarized using means and standard deviations. Comparisons between the two groups were conducted using independent sample *t*-tests or the non-parametric Mann–Whitney U test, while the Kruskal–Wallis H test was used for multi-group comparisons. Spearman’s rho correlation coefficient was employed to assess relationships between continuous variables. To evaluate the effects of graft type, extension to the external iliac artery, and graft length on dPWV, a General Linear Model (GLM) was applied, allowing for the assessment of multiple predictors and their interactions. All statistical analyses were performed using SPSS v29.0, with statistical significance set at *p* < 0.05.

## 3. Results

### 3.1. Patient Demographics and Comorbidities

During the period that this study was conducted, 146 patients with thoracoabdominal, juxtarenal, and infrarenal aneurysms were subjected to endovascular repair. A total of 39 patients were excluded according to the exclusion criteria. Table 2 presents the reasons these patients were excluded from our study. A total of 107 consecutive patients with AAAs subjected to EVAR who fulfilled the inclusion criteria were included in this study. The mean age of patients was 72 ± 7.5 years, and 93.5% of them were males.

Table 3 presents the demographics, smoking and alcohol consumption habits, and comorbidities of the patients. The mean body mass index (BMI) of patients was 27.1 ± 3.91 kg/m^2^, and 52.3% were active smokers, while the rest of the patients had quit smoking, with only two patients having never smoked in their lives. Overall, 7.5% of patients consumed alcohol on a regular basis, 41.1% consumed alcohol socially, and 51.4% did not consume alcohol at all. The most common comorbidities of patients were dyslipidemias (73.8%) and hypertension (70.1%), followed by chronic kidney disease (CKD) (68.3%), coronary artery disease (CAD) (36.4%), diabetes mellitus (19.6%), stroke (16.8%), chronic obstructive pulmonary disease (COPD) (16.8%), atrial fibrillation (AF) (12.1%), and lower extremity artery disease (LEAD) (4.7%).

### 3.2. AAA Characteristics, Endograft Properties, Operation Details

Regarding the anatomic characteristics of AAAs, the mean transverse AAA diameter of the patients was 5.7 ± 1.1 mm, the mean neck length was 18.4 ± 4.9 mm, the mean β-angle (infrarenal) was 28.8 ± 19.8°, the mean neck diameter was 24.2 ± 3.7 mm, the morphology of necks was 43.6% reversed tapered, 22.4% tapered, 27.1% straight, and 0.9% angulated, the mean iliac (sealing) diameter was 13.6 ± 4.2 mm, and the mean distance between the lowest renal artery and the right common iliac bifurcation was 175.4 ± 22 mm. The endografts implanted were Ankura (Lifetech Scientific, Shenzen, China) in seventy-three patients (68.2%), Endurant II (Medtronic Inc., Minneapolis, MN, USA) in ten patients (9.3%), Gore Excluder (W. L. Gore & Associates, Inc., Flagstaff, AZ, USA) in nine patients (8.4%), Alto (Endologix Inc., Irvine, CA, USA) in six patients (5.7%), Jotec (Jotec GmbH, Hechingen, Germany) in five patients (4.7%), and Zenith Alpha (Zenith Alpha AAA, Cook, Inc., Bloomington, Indiana) in four patients (3.7%). The mean endograft length was 169.7 ± 26.9 mm. The mean diameter of the main body of endografts deployed was 29.4 ± 4 mm, and the mean diameter of the right iliac limb of endografts deployed was 18.6 ± 5.5 mm. An extension to the right external iliac artery was deployed in 10 patients (9.3%). A total of 73 patients (68.2%) were administered general anesthesia, and 34 patients (31.8%) received regional anesthesia. The mean operation time was 96.6 ± 26 min, the mean radiation time was 7.3 ± 4.8 min, the mean radiation dose was 181.4 ± 172.4 mGy, and the mean contrast quantity used was 103.3 ± 67.5 mL. Table 4 presents the anatomic characteristics of AAAs, the properties of the endografts implanted, and the operation details of all patients included in our study.

### 3.3. Primary Outcomes

**Figure 2:**
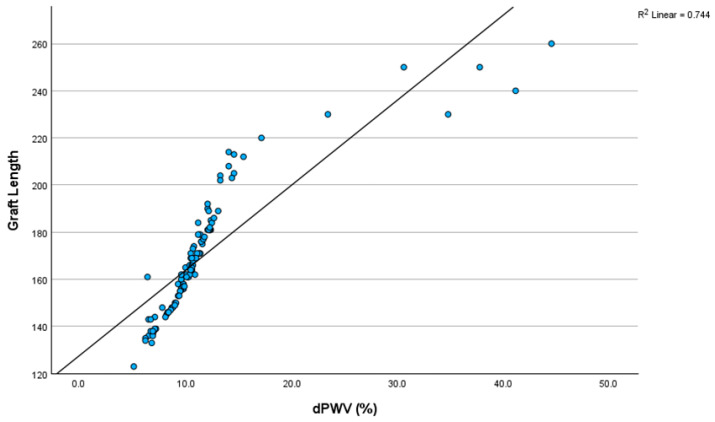
Correlation between dPWV and graft length. dPWV: change in pulse wave velocity before and after EVAR.

A strong positive correlation was observed between dPWV and endograft length, using Spearman’s rho correlation coefficient. The correlation coefficient was 0.978, indicating a strong positive correlation between graft length and the change in dPWV. The statistical significance of the correlation was *p* < 0.001. This strong positive correlation suggests that an increase in graft length is associated with a greater change in pulse wave velocity (PWV). This correlation is depicted in Figure 2.

**Figure 3:**
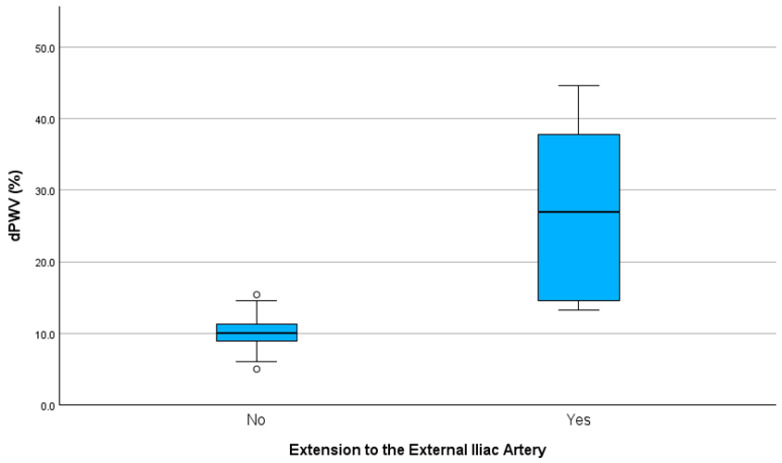
Variation in dPWV based on the presence of an extension to the external iliac artery. dPWV: change in pulse wave velocity before and after EVAR.

Significant differences in dPWV were also found based on the presence of an extension to the external iliac artery by means of an independent sample t-test. Patients without an extension had a mean dPWV of 9.95 ± 2.08%, while those with an extension exhibited a significantly higher mean dPWV of 27.12% ± 12.15%, with t = −4.463 and *p* = 0.002. This finding indicates a notable increase in arterial stiffness. This difference is visualized in Figure 3.

**Figure 4:**
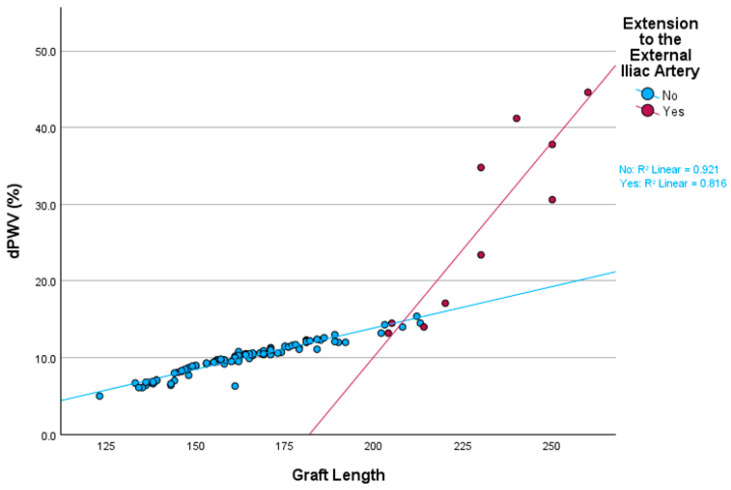
Dispersion of dPWV change in relation to graft length and extension to the external iliac artery. dPWV: change in pulse wave velocity before and after EVAR.

Multivariate analysis using GLM demonstrated a robust model fit (R^2^ = 0.942, adjusted R^2^ = 0.938), explaining 94.2% of the variance in dPWV. The presence of an extension to the external iliac artery had a statistically significant effect on dPWV (F = 171.626, *p* < 0.001, η^2^ = 0.634), with patients exhibiting significantly higher dPWV values when an extension was present (mean difference = 16.825). Extension to the external iliac artery was associated with an average increase of 90.680% in dPWV (*p* < 0.001). Graft length was also a significant determinant of dPWV (F = 319.339, *p* < 0.001, η^2^ = 0.763), with longer grafts corresponding to greater increases in dPWV. Graft length exhibited a significant positive association (B = 0.541, *p* < 0.001), indicating that each additional 1 mm in graft length corresponded to a 0.541% increase in dPWV. Figure 4 presents the dispersion of dPWV in relation to graft length and extension to the external iliac artery.

### 3.4. Secondary Outcomes

After separating the patients into three groups based on the material comprising the endograft they received, nineteen patients received a Dacron (D) endograft (Endurant, Jotec and Zenith Alpha), seventy-nine patients received an ePTFE with suprarenal fixation (Ps) endograft (Ankura, Alto), and nine patients received an ePTFE with infrarenal fixation (Pi) endograft (Gore Excluder). According to the Kruskal–Wallis test, no statistically significant differences in dPWV between these three endograft types were found (*p* = 0.74). Table 5 presents the respective findings.

**Figure 5:**
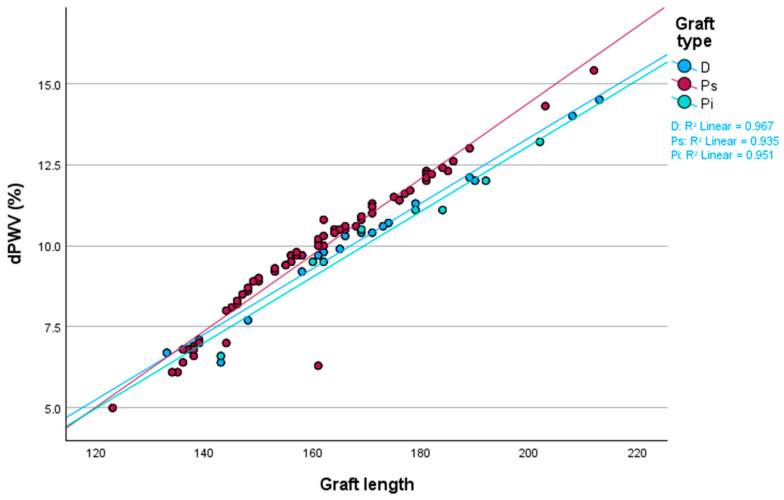
Dispersion of dPWV in relation to graft length and graft type. dPWV: change in pulse wave velocity before and after EVAR; D: Dacron polyester endograft; Ps: ePTFE endograft with suprarenal fixation; Pi: ePTFE endograft with infrarenal fixation.

Figure 5 depicts the dispersion of dPWV in relation to graft length and graft type.

No serious intraoperative complications occurred during EVAR procedures on patients included in our study. Primary technical success was achieved in 106/107 patients (99.1%). In one patient, an endoleak type Ia was recorded in completion angiography and was managed with a proximal extension deployment securing sealing, which was confirmed at the final DSA. Secondary technical success was achieved in all patients (100%). Mortality during the immediate post-operative period until discharge from hospital was 0% (0/107 patients). Minor complications (wound infection, fever, post-implantation syndrome, and urine infection) during the immediate post-operative period until discharge from hospital were recorded in 11 patients (10.2%). All minor complications subsided spontaneously or were managed successfully with no further deterioration. No clinical symptoms and signs typical of cerebral, lower limb, intestinal, spinal cord, pelvic ischemic, or hemorrhagic post-operative complications were recorded. No clinical symptoms and signs typical of pneumonia or other major respiratory complications were recorded.

## 4. Discussion

EVAR is a safe therapeutic choice for the management of AAAs, as it demonstrates low morbidity and mortality during the immediate post-operative period [3]. However, the implantation of endografts during EVAR causes an increase in aortic rigidity and, hence, aortic stiffness [5]. Aortic stiffness has been proven to be a strong independent factor in cardiovascular outcomes [11]. As a consequence, EVAR has been related to possible harmful effects on heart function and other organs, including the kidneys [12,13,14]. In the literature, various studies have investigated the correlation between various properties of endografts and the increase in aortic stiffness and PWV. Although the length of the endograft has already been positively correlated with the increase in PWV after TEVAR [15], the correlation between the treatment length of the abdominal aorta and the increase in PWV after EVAR has not been studied yet. The aim of this study was to investigate the correlation between the length of the endografts implanted during EVAR and the increase in PWV post-operatively, along with other endograft properties related to PWV and thus aortic stiffness changes post-EVAR compared to pre-operative measurements.

The measurement of PWV is principally adopted as the simplest, noninvasive, robust, and reproducible method to determine arterial stiffness. Carotid–femoral PWV is measured directly, and it corresponds to the widely accepted propagative model of the arterial system [6].

Our study found that there is a strong correlation between the length of the endografts implanted during EVAR and the increase in the PWV and, thus, aortic stiffness. This finding is in accordance with the findings of the studies regarding the positive correlation between PWV increase and the treatment length of the thoracic aorta during TEVAR. Hori et al., in their study, support the finding that an increase in PWV after TEVAR is associated with the endograft length and the device selection, while the treatment site does not affect changes in PWV [15]. Meanwhile, Yamashita et al., in their study, found that treatment length or device type were not predictors of PWV increase after TEVAR [19]. Moreover, Georgakarakos et al., studying Ovation endografts (Endologix Inc., Irvine, CA, USA) implanted in three AAA patients, did not find an increase in PWV during the early post-operative period after EVAR [20]. However, in our study, we included AAA patients treated with the Alto endograft, the new redesigned Ovation device with a sealing ring 6 mm closer to the top of the fabric, and we found that even in these patients, there was a positive correlation between endograft length and the increase in PWV.

Moreover, we found that PWV was excessively increased in cases in which the iliac limbs of endografts landed on the external iliac arteries as the sealing zone. Obviously, hemodynamics plays a major role in these cases, affecting the dramatic increase in PWV, which was related to endograft length, not with a linear correlation but with a geometrical one. Catanese et al., in their review, found that the extension of the endograft into the external iliac arteries was strongly related to limb graft occlusion after EVAR [21]. The extension of endografts to external iliac arteries, which are usually narrower than common iliac arteries, causes hemodynamic changes that could be related to adverse events regarding not only endograft patency but also other parameters, like PWV.

Although we found, in our study, that the extension of endografts to external iliac arteries is related to an excessive increase in PWV, we did not find any correlation between the different types of endograft fabrics and PWV elevation after EVAR. This finding is in contrast to Kadoglou et al.’s findings, who found that Dacron polyester recipients presented greater PWV elevation. However, Kadoglou et al., in their study, compared Dacron polyester devices, most of which had suprarenal fixation (Endurant and Talent—Medtronic Vascular, Santa Rosa, CA, USA and Zenith FB—Cook Inc., Bloomington, IN, USA) with an ePTFE endograft, which had infrarenal fixation (Gore Excluder) exclusively [22]. In our study, we included AAA patients treated with both Dacron polyester (Endurant, Jotec, and Zenith Alpha) and ePTFE with infrarenal fixation (Gore Excluder) endografts, but we also included patients treated with ePTFE with suprarenal fixation (Ankura and Alto) endografts. Even though dPWV was significantly increased in all three types of endografts, no statistically significant differences in post-operative PWV increase were found between the various types of endograft fabrics. Also, in all endograft types, an excessive increase in PWV was found when endografts were extended to the external iliac arteries.

Cumulatively, we assume based on our findings that minimum sealing lengths according to the current guidelines and instructions for use of endoprostheses should be used, avoiding unnecessary extension of iliac limbs to the common iliac bifurcations when common iliac arteries are long. Extensions to external iliac arteries should be avoided, too, whenever this is possible. Regarding the choice of endoprosthesis material for EVAR, endoprostheses made from materials causing a lower possible increase in PWV and thus aortic stiffness, affecting native wall compliance to the minimum possible degree, should be preferred.

Regarding the limitations of our study, our study is a monocentric, single-arm observational study with a relatively small sample size. Although our results could be presumed reliable according to power analysis, generalization of the results should be avoided, as observational studies present a lower level of evidence compared to randomized ones. The patients recruited in our study are sufficiently homogenous, as they were subjected to EVAR with aortobiiliac endografts meeting certain inclusion criteria. On the contrary, these narrower criteria applied in order to gather a homogeneous group of patients have as a consequence the rejection of patients subjected to ChEVAR or BEVAR. Separating the patients into the three endograft material groups (D, Ps, and Pi) and including patients with endografts from different companies in each group could lead to biased results. However, this classification, which has already been used in other studies in the literature, allowed us to extract some deductions regarding the effect of the endograft material on PWV. Moreover, we measured PWV during the immediate post-operative period after EVAR, while the negative effects of endografts regarding PWV changes and organ injuries could be seen even after months or years. These outcomes are beyond the purposes of the present study, but they warrant further investigation in future studies. The investigation of ameliorating PWV increase and thus aortic stiffness by improving various properties of the endografts could contribute to minimizing the endograft’s impact on the native aortic wall and probably prevent adverse cardiovascular events in EVAR patients.

## 5. Conclusions

This prospective, monocentric, single-arm observational study suggests that treatment length, which practically means the endograft length, affects PWV elevation to different degrees during the immediate post-operative time in EVAR patients, especially when the endograft is extended to external iliac arteries. PWV increase and aortic stiffness are strong independent factors of cardiovascular outcomes. The improvement in endograft properties used in AAA patients subjected to EVAR could minimize aortic stiffness, which is caused by the implantation of endografts, leading to the prevention of adverse cardiovascular events. Understanding the relationship between endograft length, extension to external iliac arteries, and PWV elevation is crucial for optimizing EVAR procedures and potentially mitigating adverse cardiovascular outcomes associated with increased aortic stiffness. Further multicentric studies with larger sample sizes are needed to validate our findings and explore the long-term impact of endograft properties on cardiovascular outcomes post-EVAR.

## Figures and Tables

**Table 1:** Inclusion and exclusion criteria.

Inclusion Criteria	Exclusion Criteria
Adult patients (males or females)	Ruptured, inflammatory, or mycotic AAA
AAA affecting the infrarenal aorta exclusively	Complex aortic aneurysms
AAA threshold diameter for EVAR of ≥5.5 cm in men or ≥5 cm in women	ChEVAR, BEVAR, FEVAR, or EVAR with aortouniiliac devices or straight tube endografts
EVAR electively conducted	Connective tissue disorders
Aortobiiliac endografts (with external artery extension if needed)	End-stage renal disease
	Severe atherosclerotic disease with severe hemodynamic changes in arteries could bias PWV measurement
	Operations (open and/or endovascular) on arteries could bias PWV measurement
	Previous operations on the aorta (open and/or endovascular)

AAA: abdominal aortic aneurysm; EVAR: endovascular aortic repair; ChEVAR: chimney endovascular aortic repair; FEVAR: fenestrated endovascular aortic repair; BEVAR: branched endovascular aortic repair; PWV: pulse wave velocity.

**Table 2:** Patients excluded from this study.

Exclusion Criteria	Patients (n)
Ruptured AAAs	12
Aortic aneurysm managed with BEVAR/FEVAR	6
Aortic aneurysm managed with ChEVAR	9
End-stage renal disease	2
Severe atherosclerotic disease with severe hemodynamic changes in arteries could bias PWV measurement	4
Operations (open and/or endovascular) on arteries could bias PWV measurement	3
Previous repairs of the aorta (open and/or endovascular)	3
**Total**	39

n: number; AAA: abdominal aortic aneurysm; BEVAR: branched endovascular aortic repair; FEVAR: fenestrated endovascular aortic repair; ChEVAR: chimney endovascular aortic repair; PWV: pulse wave velocity.

**Table 3:** Patient demographics and comorbidities.

Demographic, Comorbidity	Finding
Number of patients, n	107
Males:females, n:n (%:%)	100 (93.5%): 7 (6.5%)
Age, years (±SD)	72 (±7.5)
BMI, kg/m^2^ (±SD)	27.1 (±3.91)
Smokers: Active, n (%)	56 (52.3)
Previous, n (%)	49 (45.8)
Never, n (%)	2 (1.9)
Alcohol: Regular basis, n (%)	8 (7.5)
Socially, n (%)	44 (41.1)
No, n (%)	55 (51.4)
Hypertension, n (%)	75 (70.1)
Dyslipidemias, n (%)	79 (73.8)
CAD, n (%)	39 (36.4)
ACS, n (%)	23 (21.5)
PCI, n (%)	26 (24.3)
CABG, n (%)	9 (8.4)
HF, n (%)	2 (1.9)
Diabetes mellitus, n (%)	21 (19.6)
COPD, n (%)	18 (16.8)
CKD, n (%)	73 (68.3)
I stage, n (%)	31 (29)
II stage, n (%)	29 (27.1)
III stage, n (%)	11 (10.3)
IV stage, n (%)	2 (1.9)
AF, n (%)	13 (12.1)
Stroke, n (%)	18 (16.8)
Carotid disease (left >50% stenosis), n (%)	4 (3.7)
LEAD, n (%)	5 (4.7)

n: number; SD: standard deviation; BMI: body mass index; CAD: coronary artery disease; ACS: acute coronary syndrome; PCI: percutaneous coronary intervention; CABG: coronary artery bypass graft surgery; HF: heart failure; COPD: chronic obstructive pulmonary disease; CKD: chronic kidney disease; AF: atrial fibrillation; LEAD: lower extremity arterial disease.

**Table 4:** AAA characteristics, endograft properties, and operation details.

Anatomic Characteristics of AAAs	Finding
Mean transverse diameter, mm (±SD)	5.7 (±1.1)
Mean neck length, mm (±SD)	18.4 (±4.9)
Mean β-angle, ^o^ (±SD)	28.8 (±19.8)
Mean neck diameter, mm (±SD)	24.2 (±3.7)
Neck morphology: Reversed tapered, n (%)	53 (43.6)
Tapered, n (%)	24 (22.4)
Straight, n (%)	29 (27.1)
Angulated, n (%)	1 (0.9)
Mean iliac (sealing) diameter, mm (±SD)	13.6 (±4.2)
Mean distance between the lowest renal artery to the right common iliac bifurcation, mm (±SD)	175.4 (±22)
**Endograft properties**	**Finding**
Company: Ankura, n (%)	73 (68.2)
Endurant II, n (%)	10 (9.3)
Gore Excluder, n(%)	9 (8.4)
Alto, n (%)	6 (5.7)
Jotec, n (%)	5 (4.7)
Zenith Alpha, n (%)	4 (3.7)
Mean endograft length, mm (±SD)	169.7 (±26.9)
Mean diameter of main body, mm (±SD)	29.4 (±4)
Mean diameter of right iliac limb, mm (±SD)	18.6 (±5.5)
Extension to right external iliac artery, n (%)	10 (9.3)
**Operation details**	**Finding**
Anesthesia: General, n (%)	73 (68.2)
Regional, n (%)	34 (31.8)
Mean operation time, min (±SD)	96.6 (±26)
Mean radiation time, mm (±SD)	7.3 (±4.8)
Mean radiation dose, mGy (±SD)	181.4 (±172.4)
Mean contrast quantity, ml (±SD)	103.3 (±67.5)

AAA: abdominal aortic aneurysm; mm: millimeter; n: number; SD: standard deviation; o: degrees; min: minutes; mGy: milligray; ml: milliliters.

**Table 5:** Kruskal–Wallis test for dPWV difference between the three endograft groups.

Endograft Type	Ν	Median	IQR	K-W	*p*
D	19	10.40	2.10	0.602	0.740
Ps	79	10.30	3.05		
Pi	9	11.10	2.50		

D: Dacron polyester endograft; Ps: ePTFE endograft with suprarenal fixation; Pi: ePTFE endograft with infrarenal fixation; IQR: interquartile range; K-W: Kruskal–Wallis test.

## Data Availability

All details about the study are included in the present manuscript.

## Abbreviations

The following abbreviations are used in this manuscript:

AAA	Abdominal aortic aneurysm
CT	Computed tomography
OSR	Open aortic repair
EVAR	Endovascular aortic repair
ePTFE	Expanded polytetrafluoroethylene
PWV	Pulse wave velocity
dPWV	Change in pulse wave velocity before and after EVAR
CTA	Computed tomography angiogram
DSA	Digital subtraction angiography
D	Dacron endograft
Ps	ePTFE endograft with suprarenal fixation
Pi	ePTFE endograft with infrarenal fixation
ChEVAR	Chimney endovascular aortic repair
FEVAR	Fenestrated endovascular aortic repair
BEVAR	Branched endovascular aortic repair
LMWH	Low-molecular-weight heparin
BMI	Body mass index
CAD	Coronary artery disease
ACS	Acute coronary syndrome
PCI	Percutaneous coronary angioplasty
CABG	Coronary artery bypass graft surgery
HF	Heart failure
COPD	Chronic obstructive pulmonary disease
CKD	Chronic kidney disease
AF	Atrial fibrillation
LEAD	Lower extremity arterial disease
TEVAR	Thoracic endovascular aortic repair
SD	Standard deviation
IQR	Interquartile range
K-W	Kruskal–Wallis test
GLM	General linear model
